# The epidemiological and economic impact of a quadrivalent human papillomavirus (hpv) vaccine in Estonia

**DOI:** 10.1186/1471-2334-13-304

**Published:** 2013-07-03

**Authors:** Anneli Uusküla, Andres Müürsepp, Kosuke Kawai, Mait Raag, Mikk Jürisson, Matthew Pillsbury

**Affiliations:** 1 Department of Public Health, University of Tartu, Ravila st 19, Tartu 50411, Estonia; 2 Merck Sharp & Dohme OÜ, Tallinn, Estonia; 3 Temple University, Philadelphia, PA 19140, USA; 4 East Tallinn Central Hospital, Tallinn, Estonia; 5 Atlas Data Systems, Westfield, NJ, USA

**Keywords:** HPV, Vaccine, Cost effectiveness, Screening, Cervical cancer, Genital warts, Estonia

## Abstract

**Background:**

This analysis assessed the epidemiological and economic impact of quadrivalent human papillomavirus (HPV4: 6/11/16/18) vaccination in Estonia.

**Methods:**

A dynamic transmission model was used to assess the epidemiological and economic impact of the routine vaccination of 12-year-old girls with a HPV4 vaccine in preventing cervical cancer, cervical intraepithelial neoplasia (CIN) grades 1, 2 and 3 and genital warts.

**Results:**

The model projected that at year 100, HPV4 vaccination would lead to a reduction of HPV 16/18 related cervical cancer incidence and deaths by over 97% and the incidence of HPV 6/11 related genital warts among Estonian women and men by over 94% and 81%, respectively. The incremental cost-effectiveness ratio of the HPV4 vaccination strategy was € 4,889 per QALY gained over a time horizon of 100 years.

**Conclusions:**

Routine vaccination of 12-year-old girls with HPV4 vaccine appears to be cost-effective in Estonia, in addition to providing both short term and long term health gains.

## Background

Evaluations of the cost-effectiveness of high-risk human papillomavirus (HPV) vaccination as a cervical cancer prevention measure are mostly limited to countries in Western Europe and North America [1]. Few studies have assessed the cost effectiveness of HPV vaccination in Central and Eastern European countries [2-5]. Countries in this region tend to have less-developed cervical cancer screening programs, and subsequently have considerably higher age-standardized cervical cancer incidence rates (14.7 per 100,000, 2008) than those in Western Europe (6.9 per 100,000, 2008) or North America (5.7 per 100,000, 2008) [6].

In Estonia the cervical cancer incidence (age-standardized incidence rate 15.8 per 100,000, 2008) and mortality (age-standardized mortality rate 6.2 per 100,000, 2008) are significantly higher than in many developed countries including neighboring Scandinavian countries [6]. Current coverage with any type (opportunistic or systematic) of cervical cytology (PAP smear) testing-based cervical cancer screening is relatively high (3-year coverage of ~72%) in the country [7]. Systematic screening implemented in parallel with ongoing opportunistic screening was introduced in 2006 [8] but has achieved only limited coverage (24% of the target population, women aged 30 to 59 years in 2009). Both bivalent and quadrivalent HPV (HPV2 and HPV4) vaccines are approved for use in Estonia [9].

This study investigated the clinical benefits and economic consequences of routine quadrivalent HPV vaccination of females by the age of 12 years in Estonia. Specifically, this study was designed to evaluate the potential impact in Estonia of prophylactic quadrivalent HPV vaccination on the incidence of cervical intraepithelial neoplasia (CIN), cervical cancer, genital warts, and cervical cancer mortality when added to the current cervical cancer screening and standard of care.

## Methods

We adapted a previously developed HPV dynamic mathematical model to Estonia (Elbasha & Dasbach, 2010: [10]). Details of the model structure and equations have been published previously [10]. Individuals enter the model as they are born, move between successive age groups at an age- and gender-specific rate per year, and exit the model as they die. The model estimates health benefits and costs in a dynamic population. The model also estimates the impact of vaccination on vaccinees and their contacts (via herd immunity impact).

### Demographic and epidemiological model

The model simulated aging and all-cause mortality over time within the Estonian population. The model simulated the transmission of HPV infection within the population as determined by the course of sexual mixing, a feature which allows for estimating both the direct and indirect (i.e. herd immunity) benefits of vaccination. Hence, the model required inputs on sexual activity risk groups in the population.

### Vaccination and screening strategies

In the model, it is assumed that vaccination occurs prior to sexual debut and would consist of the three recommended doses, and the vaccination would confer type-specific protection. Other parameters subjected to greater uncertainty such as vaccination coverage and duration of protection were further explored in the sensitivity analysis. The model incorporates vaccine efficacy from the most recent clinical trials. The prophylactic vaccine efficacy against transient HPV 6, 11, 16 and 18 infections was assumed to be 76.1%, 76.1%, 76.0%, and 96.3%, respectively (Merck & Co., Inc. Unpublished data 2009). The vaccine efficacy against persistent HPV 16 and 18 infections was assumed to be 98.8% and 98.4%, respectively.

In both the vaccinated and unvaccinated cohorts, screening was modelled according to the actual current/routine Estonian cervical cancer screening practice.

### Model inputs: values and sources

The model requires input values for demographic, behavioural, epidemiological, screening, treatment, vaccine, and economic parameters (see Additional file 1: Tables A1-A13).

When available, we used data from Estonia as inputs, otherwise we used the default data in the model published for the US [10] (inputs not presented in this report are available in the online supplement of Elbasha & Dasbach, 2010 [10]).

The Estonian Health Insurance Fund (HIF) database was used as the source for health care utilization data, HPV disease occurrence and health care costs (see Additional file 1: Tables A1-A13). Health insurance in Estonia is funded through a compulsory scheme under which employers are obliged by law to pay social and health insurance taxes for their employees. Self-employed people pay a social tax based on their income. Individuals whose social/health insurance tax is paid by their employer or who pay it themselves are considered to be covered by health insurance ("the insured") and are members of the HIF. As of 31 December 2008, 1,281,718 people were registered as insured by the HIF representing 95.6% of the Estonian population [11]. The HIF database is a “reimbursement database” containing information on ambulatory and in-patient/hospital care as well as reimbursed pharmaceuticals. As HIF reimburses health care providers on a fee-for-service basis and, the database is considered to be relatively complete.

Data sources:

1) Demographic data (population size and composition, age and gender specific mortality) was taken from Statistics Estonia (Statistics Estonia, [12]) (Table A1);

2) Sexual behaviour data was derived from the Estonian Health Interview Survey 2006 [13]. Estimates on the mean number of sexual partners per year by gender and age group were used and categorised as: low (0–1 per year), medium (2–4 per year), and high (5+ per year). The parameter was modified for model calibration purposes. The data on mean partners by age categories were changed so that the model projected a close estimate of the observed cervical cancer incidence and deaths in the Estonian population (Tables A3, A4 and section on Validation Analyses for more details);

3) The age and stage-specific cervical cancer mortality was assumed to be the same as in the US [14] (Table A2);

4) Screening data (cervical cancer screening rate by age group, % per year, proportion of women with a follow-up screening following an abnormal PAP) (Table A5);

5) Treatment variables (Table A5):

(i) proportion of women with cervical cancer who develop symptoms and seek care, by cancer stage – Estonian cancer registry;

(i) proportion of CIN/carcinoma in situ (CIS) treated, by stage – expert opinion and literature [10]. Based on local data (expert opinion), it was assumed that 50% of diagnosed CIN1 cases and 100% of diagnosed CIN2/CIN3 cases receive treatment; and

(i) hysterectomy rates by age group, % per year – based on the data from HIF;

6) Economic variables:

(i) direct medical costs of interventions were estimated using the national tariffs of the HIF for 2011 were reported in euros. Euro is the national currency in Estonia.

(i) costs of diagnosing and treating HPV disease (genital warts, cervical cancer screening and visit, colposcopy, biopsy, CIN 1,2,3 episode-of-care, cervical cancer (local, regional, distant) episode of care) – data from HIF (Table A6; A8—A13);

(i) vaccine cost: A cost of €59.00 per dose for the HPV4 vaccine was used, based on assumptions made in an earlier published analysis for Estonia [15] (Table A4)

7) Vaccine strategy variables are based on the following assumptions and data sources:

(i) HPV4 coverage of 85% of females age 12. This was set slightly below the 91.2% reported in 2010 for coverage with two doses of measles, mumps, and rubella vaccine (MMR), the second dose of which is administered to 13- to 14-year-old adolescents within the state calendar vaccination programme and via the school health system [16];

(i) 100% adherence with the 3-dose regimen;

(i) Vaccine will be delivered through the existing school-based delivery system, similar to the current MMR regimen;

(i) Lifetime duration of protection was assumed in base scenarios. Effects of different lengths of duration were tested in a sensitivity analysis.

### Cost effectiveness analysis

To calculate the cost-effectiveness of the vaccination strategy in preventing disease with respect to costs, we used the total discounted costs and effects (i.e. quality-adjusted life years, QALYs) accrued over a 100-year period with and without vaccination. In addition, the incremental costs incurred to achieve the incremental benefits by vaccination were calculated and the ratio of the incremental costs to incremental QALYs gained (i.e., the incremental cost-effectiveness ratio, ICER) are presented.

QALYs were estimated based on health utilities from the U.S. (Table A7). In addition to utilities for HPV disease states, age and gender-specific utility weights were also incorporated for individuals without HPV disease to account for the impact of co-morbid conditions (Table A7).

Both costs and medical outcomes were discounted at an annual rate of 3% [17,18].

### Model validation

As described above, the model was calibrated for Estonia using sexual activity and cancer detection rate parameters. We assessed the predictive validity of the model by comparing model predictions with observed data on the age specific incidence of cervical cancer and cervical cancer deaths in Estonia.

### Sensitivity analyses

The cost effectiveness analysis is based on a number of assumptions. Due to uncertainty in some of these assumptions, several one-way sensitivity analyses were carried out. The parameters included duration of vaccine protection (20 years), vaccine coverage rates (70%, 95%), HPV disease diagnosis and treatment costs (+/−20%), vaccine cost per dose (+/−10%), discount rate (5%), and QALY weights. We also examined a scenario assuming no quality of life adjustments (cost per life years saved). Additionally, we examined the impact of HPV 6 and 11 protection in HPV4 on cost-effectiveness by running a scenario without the HPV 6 and 11 protection.

We also examined the cost-effectiveness of HPV vaccination under a hypothetical improved screening program. This hypothetical program was assumed to have immediate coverage of 95% (compared with the current/routine coverage of 72%) and the same age-specific annual rate as the base case scenario. Hypothetical improved screening independently gradually reduced the HPV16/18-related cervical cancer incidence to ~6 per 100,000 per year (age-standardized) at the steady-state in the model, about 100 years after screening had been initiated in the population. This steady-state cervical cancer incidence corresponded to ~ 8 per 100,000 per year of any HPV type (based on the assumption of 76% contribution of HPV 16/18 to cervical cancer; [6]) and mirrored the current cervical cancer incidence observed in the Western Europe [19].

## Results

### Model validation

The overall incidence of HPV type-16/18-related cervical cancer projected from the model was 11.8 per 100,000 females and cervical cancer mortality was 3.4 per 100,000 females. This is consistent with the overall cervical cancer incidence of 15.8 and mortality of 4.7 per 100,000 females [6], of which approximately 76% (i.e. 12.1 and 3.6, respectively) is thought to be HPV16/18-related [6].

The projected incidence of HPV 6/11-related genital warts (GW) treated was about 110 cases per 100,000 for females and 45 per 100,000 for males. The GW treatment incidence differed by gender. We incorporated this gender difference in the model by changing the proportion of those who received treatment among those who developed GW. This is consistent with overall GW treatment incidence of 123/50 (females/males) per 100,000 (HIF database – ICD-10 diagnosis code A63.0, average annual incidence of 7 years over period of 2004–2010), of which approximately 90% (i.e. 111/45, females/males respectively, per 100,000) is thought to be related to types 6/11 [10].

### Clinical / health effects

#### Annual incidence of disease cases

**Figure 1:**
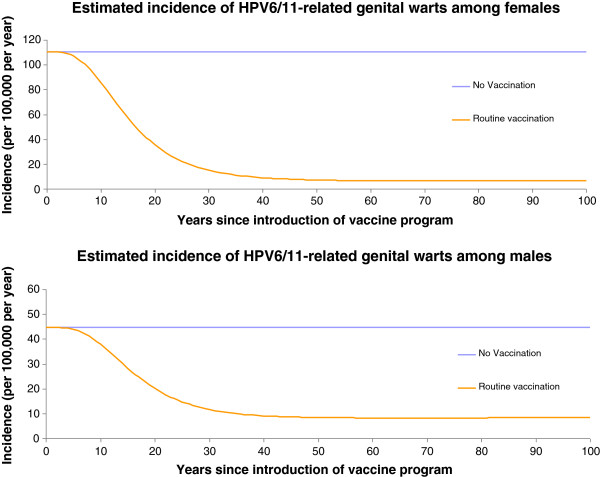
Incidence of HPV 6/11 related genital warts among men and women per 100 000 population over time, Estonia.

**Figure 2:**
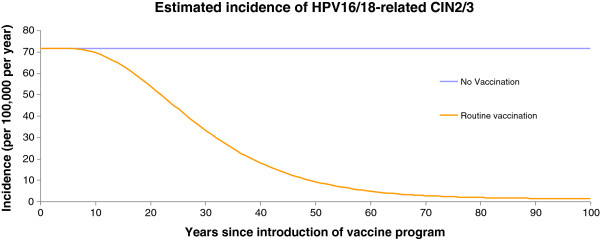
Incidence of HPV 16/18-related CIN 2/3 per 100 000 population over time, Estonia.

**Figure 3:**
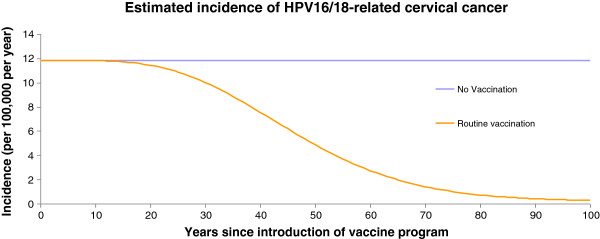
Incidence of HPV 16/18-related cervical cancer per 100 000 population over time, Estonia.

Figures 1, 2, and 3 present the changes in occurrence of genital warts (among women and men), CIN2, 3, and cervical cancer incidence, respectively.

The effect of vaccination was to progressively reduce the incidence of cervical cancer cases and deaths until the system approached a steady state, about 100 years after vaccination has been initiated in the population. Compared with no vaccination, the vaccination reduced the incidence of 16/18-related cervical cancer cases and deaths by over 97% by year 100. At year 50, the vaccination strategies reduced the incidence of 16/18-related cervical cancer cases by 59% (no vaccination vs vaccination: 11.8 vs 4.85 per 100 000), and deaths by 50% (no vaccination vs vaccination: 3.42 vs 1.71 per 100 000). Numbers of HPV 16/18-related cervical cancer cases were halved in the vaccinated group by year 46.

A similar dynamic was observed for the reductions in CIN 2/3, CIN 1, genital warts-female and genital warts-male cases as displayed in Figures 1 and 2. These curves share similar qualitative features with those of cervical cancer, but are shifted to the left compared with the cervical cancer curves (Figure 3).

At year 100, the model predicted that the vaccination strategy would provide over 98% relative reduction in the incidence of 6/11/16/18-related CIN 2/3 and CIN 1, and 94% and 81% reductions in HPV 6/11 related genital warts in females and males, respectively. The number of 6/11/16/18-related cases of CIN2/3, CIN1, genital warts-female, and genital warts-male were halved by year 29, 26, 16, and 19.

#### Cumulative impact in population over 100 years

Adding vaccination to the cervical cancer screening led to significant health benefits. The model showed reductions over 100 years in: HPV 16/18 related deaths (50%); cervical cancer incidence (52%); CIN 2/3 (68%); and genital wart incidence among women (79%) and men (67%) (over 100 years) (Table 1).

**Table 1:** Cumulative reduction in cases (percent) of HPV 6/11/16/18 disease by HPV vaccination over time in Estonia, model estimates

	**Over 25 years**	**Over 50 years**	**Over 100 years**
HPV16/18-related cervical cancer	31 (2%)	654 (17%)	4103 (52%)
HPV16/18-related CIN2/3	1390 (12%)	9541 (40%)	32508 (68%)
HPV16/18-related CIN1	554 (15%)	3391 (45%)	10760 (71%)
HPV6/11-related CIN1	271 (32%)	1016 (61%)	2591 (77%)
HPV6/11-related genital warts (female)	6663 (36%)	23246 (63%)	58035 (79%)
HPV6/11-related genital warts (male)	2092 (28%)	7873 (53%)	20047 (67%)

* Percentages rounded to nearest 1; ** Cases rounded to nearest 1.

#### Economic impact and cost effectiveness analysis

Introduction of vaccination program will lead to a 29% reduction of disease costs over 100 years. Per person discounted costs and effects (i.e., QALYs) accrued over a 100-year period with and without vaccination strategy, incremental costs, and incremental QALYs, and the incremental cost effectiveness ratio are presented Table 2. The incremental cost-effectiveness ratio (ICER) for routine vaccination of girls by age 12 was €4,889/QALY.

**Table 2:** Cost effectiveness of HPV vaccination in Estonia, model estimates

	**Discounted total**		**Incremental**		
	Costs/person (€)*	QALY/person (year)**	Costs/person (€)*	QALY/person (year)**	Costs/QALYs (€/year)***
No vaccination	126.29	27.09184	--	--	--
Routine vaccination	152.79	27.09726	26.5	0.00542	4889

*Costs rounded to 0.01; **QALYs rounded to 0.00001; ***Costs/QALYs rounded to 1.

#### Sensitivity analysis

The ICER increased when we excluded protection against HPV 6/11 disease (€ 5771 per QALY) or limited the time of vaccine protection to 20 years (€ 8090 per QALY) (Table 3). The ICER was € 6167 per QALY under the assumption of hypothetical (improved) cervical cancer screening in Estonia. The rate of discounting was one of the influential factors affecting the results. The ICERs did not vary by the changes to health care costs related to HPV disease diagnosis and treatment.

**Table 3:** Sensitivity analysis: incremental cost-effectiveness ratio (ICER) of HPV vaccination* in Estonia, model estimates.

	**ICER € per QALYs**
Base case	4889
*Screening characteristics*	
Under hypothetical improved cervical screening program**	6167
*Vaccine characteristics*	
Assuming no HPV 6/11 protection	5771
Duration of protection = 20 years	8090
*Vaccination characteristics*	
Low vaccine coverage (70%)	4444
High vaccine coverage (95%)	5212
*Costs characteristics*	
Cost of diagnosis and treatment +20%	4744
Cost of diagnosis and treatment −20%	5037
Cost of vaccine - 10%	4351
Cost of vaccine + 10%	5454
*Health utility values*	
Cost per life year saved (no quality of life adjustments)	6746
*Discount rate*	
Discount rate 0%	1517
Discount rate 5%	11148

* HPV6/11/16/18 related cervical cancer and genital warts.

**Vaccination program introduced alongside hypothetical improved cervical screening program.

## Discussion

This is one of the first studies to examine the potential impact of HPV2 and 4 vaccination in Estonia [15]. The incremental cost-effectiveness ratio of the vaccination compared with no vaccination was €4,889 per QALY gained over a time horizon of 100 years. Given that base case as well as all scenarios tested in sensitivity analysis remained below one GDP per capita in Estonia (2011: €11,900 [20]) HPV vaccination can be deemed cost effective (based on WHO recommendations on cost effectiveness [21]) in Estonia.

The analysis of HPV vaccination by Liiv et al. [15] derived a cost effectiveness estimate of €10,019 /QALY (at the 3% discount rate). Variations in the ICER estimates between the current work and that of Liiv et al. are probably explained by the different models used (dynamic transmission model incorporating the effect of herd protection vs Markov model) and several differences in the assumptions made (non-life-long immunity and booster dose inclusion; higher (95%) vaccine coverage; lower proportion (70%) of HPV 16/18 related cervical cancer contribution in Liiv et al.). Such factors are informative for contextualizing the results.

However, both studies are in agreement with the recent systematic review [1] in concluding that while different model structures, input parameters and baseline assumptions have been used to assess the cost effectiveness of HPV vaccination, studies that focused on female-only vaccination programmes usually find this intervention to be cost effective compared with cervical cancer screening alone [1].

Our results document significant health effects related to the combined use (HPV4 vaccination + cervical cancer screening) of cervical cancer prevention methods. The model projected that at year 100, HPV4 vaccination would lead to a relative reduction of HPV 16/18 related deaths and cervical cancer incidence by over 97% and the incidence of HPV 6/11 related genital warts among Estonian women and men by over 94% and 81%, respectively. In addition, there are relevant immediate public health impacts of HPV4 vaccination on genital warts among both women and men. Every year, approximately 751 women and 276 men receive treatment for genital warts in Estonia (based on HIF treatment statistics during 7 years from 2004–2010, ICD 10, diagnosis code A63.0, data not shown). Our model projected a substantial reduction in the incidence of genital warts in the first 25 years.

Based on the sensitivity analysis, the incremental costs per QALY are about 20% higher if vaccination is implemented against a background of improved cervical cancer screening. We found that the cost-effectiveness ratio was heavily impacted by the discount rate and health utility values used. Assuming a limited duration of vaccine protection and reducing the costs of the vaccine led to modest changes in ICER in opposite directions (i.e. limiting duration increased ICER and reducing costs decreased ICER).

Our findings should be interpreted with due consideration for a number of limitations. First, we did not conduct an in-depth literature review to obtain data on the natural history of site- and type-specific HPV infection and disease or on health utility requirements of HPV disease states. Instead, we abstracted relevant data from published studies [10] and we also assessed the cost per life year saved with no quality of life adjustments in the sensitivity analysis. However, for most of the key parameters Estonian specific data were used, and the model was calibrated using cervical cancer incidence/mortality in Estonia. Second, our model is based on a number of efficacy assumptions regarding transient and persistent infection which are not completely available in the published literature. Third, we did not incorporate additional potential benefits of protecting against HPV diseases such as vulvar, vaginal, and anal dysplasia/cancers in this analysis. Data from clinical trials supports the efficacy of the vaccine against these diseases [22-24]. This potentially underestimates the benefits of vaccination. On the other hand, the duration of vaccine protection is uncertain (although we assumed life-long vaccine protection in the base case). Further, the model simulates only the heterosexual transmission of HPV and does not incorporate transmission between homosexual and heterosexual individuals. Only direct medical costs were included in the model, ignoring potential savings in or outside the health care sector. Excluding indirect costs from the analysis most likely leads to undermining of the vaccination effect, given that preventing the disease (cervical cancer) adds to the productive activity. Importantly, the model could not distinguish systematic from opportunistic cervical cancer screening. Systematic screening is generally more effective and cost-effective than opportunistic screening: countries that have organised screening programmes have much lower lifetime testing and subsequent treatment rates than those with only opportunistic screening [25-27]. However, we used sensitivity analysis to evaluate the potential effect of vaccination in combination with the hypothetical/improved (systematic) screening. Our finding of a higher ICER value in the case of hypothetical/improved screening is in agreement with the current understanding of HPV disease and prevention (improved screening results in a lower incidence of cervical cancer, therefore a higher ICER for vaccination strategy). Last but not least, our sensitivity analyses were limited to the inputs related to vaccine properties and coverage, US based health utilities, health care (diagnosis, treatment) costs, and discount rate and did not include all demographic, behavioural, clinical, and other natural history parameters. This was done mainly to manage complexity in the analysis and obtain results in a timely manner for policy evaluations. As a result, the uncertainty results do not reflect the full range of possible factors.

Finally, there are several unresolved issues for successful implementation of cervical cancer prevention, including delineating the optimum set of components (primary prevention, screening, vaccination) to reduce cervical cancer incidence and mortality. Mathematical modelling studies and statistical evidence synthesis are methods that are best suited to investigating the optimal combination of different cervical cancer prevention strategies and should be developed to guide policy decisions about cancer control.

## Conclusions

In general, routine vaccination of 12-year-old females appears to be cost-effective in Estonia and may provide both short-term and long-term health gains.

## Competing interests

AM is employed by MSD. MJ is a former employee of MSD. KK is a post-doctoral fellow funded by Merck & Co., Inc. MP is a consultant to Merck & Co., Inc.

## Authors’ contributions

AU, AM, and MJ developed the concepts for and designed the country level data acquisition and analysis plans; MR organized and analysed the data; and KK and MP conducted the modeling analysis. AU wrote the first draft of the manuscript. All authors contributed to revising the manuscript critically for important intellectual content and have approved the final manuscript.

## Pre-publication history

The pre-publication history for this paper can be accessed here:

http://www.biomedcentral.com/1471-2334/13/304/prepub

## Supplementary Material

Additional file 1 **Appendix: Table A1.** All-Cause Mortality, Estonia, 2009. **Table A2**. Age and stage-specific cancer mortality (% per year). **Table A3**. Mean number of sexual partners per year by age groups, Estonia. **Table A4**. Distribution of sexual activity risk groups and corresponding mean numbers of partners per year, Estonia. **Table A5**. Screening and Treatment Parameters, Estonia. **Table A6**. Costs of diagnosing and treating HPV disease in Estonia (in €). **Table A7**. Quality-of-Life Parameters*, Estonia. **Table A8**: Diagnosis and treatment costs of genital warts – male and female, Estonia*.***Table A9**: Costs related to the conventional cytology screening exam, Estonia. **Table A10**: Costs related to colposcopy, Estonia. **Table A11**: Costs related to biopsy, Estonia. **Table A12**: Diagnosis and treatment costs assumed for CIN1, CIN 2/3, Estonia. **Table A13**: Assumed diagnosis and treatment costs of localized cervical cancer (LCC), regional cervical cancer (RCC) and distant cervical cancer (DCC), Estonia. Click here for file
