# Supplementary material for: The epidemiological and economic impact of a quadrivalent human papillomavirus (hpv) vaccine in Estonia
Source: BMC Infect Dis. 2013 Jul 3;13:304. doi: 10.1186/1471-2334-13-304 (PMC3706227; doi:10.1186/1471-2334-13-304)
Supplement: Additional file 1 — Appendix: Table A1. All-Cause Mortality, Estonia, 2009. Table A2. Age and stage-specific cancer mortality (% per year). Table A3. Mean number of sexual partners per year by age groups, Estonia. Table A4. Distribution of sexual activity risk groups and corresponding mean numbers of partners per year, Estonia. Table A5. Screening and Treatment Parameters, Estonia. Table A6. Costs of diagnosing and treating HPV disease in Estonia (in €). Table A7. Quality-of-Life Parameters*, Estonia. Table A8: Diagnosis and treatment costs of genital warts – male and female, Estonia.Table A9: Costs related to the conventional cytology screening exam, Estonia. Table A10: Costs related to colposcopy, Estonia. Table A11: Costs related to biopsy, Estonia. Table A12: Diagnosis and treatment costs assumed for CIN1, CIN 2/3, Estonia. Table A13: Assumed diagnosis and treatment costs of localized cervical cancer (LCC), regional cervical cancer (RCC) and distant cervical cancer (DCC), Estonia. [file 1471-2334-13-304-S1.docx]

**Additional file**

**Table A1. All-cause mortality, Estonia, 2009 ***

|  | All cause mortality rate | |
| --- | --- | --- |
| Age | Female | Male |
| <1 year | 0.00082 | 0.00103 |
| 1-8 years | 0.00026 | 0.00006 |
| 9-10 years | 0.00003 | 0.00019 |
| 11-12 years | 0.00003 | 0.00019 |
| 13-14 years | 0.00003 | 0.00019 |
| 15-17 years | 0.00017 | 0.00050 |
| 18 years | 0.00017 | 0.00050 |
| 19 years | 0.00017 | 0.00050 |
| 20-24 years | 0.00055 | 0.00140 |
| 25-26 years | 0.00050 | 0.00270 |
| 27-29 years | 0.00050 | 0.00270 |
| 30-34 years | 0.00043 | 0.00300 |
| 35-39 years | 0.00113 | 0.00283 |
| 40-44 years | 0.00138 | 0.00404 |
| 45-49 years | 0.00245 | 0.00727 |
| 50-54 years | 0.00367 | 0.01253 |
| 55-59 years | 0.00566 | 0.01895 |
| 60-64 years | 0.00857 | 0.02545 |
| 65-69 years | 0.01237 | 0.03652 |
| 70-74 years | 0.02105 | 0.05188 |
| 75-79 years | 0.03823 | 0.07528 |
| 80-84 years | 0.07093 | 0.10986 |
| ≥85 years | 0.15894 | 0.18744 |

* Source: Statistics Estonia, all cause mortality rate, 2009 ([www.stat.ee](http://www.stat.ee), accessed last 01-Oct-2011)**Table A2. Age and stage-specific cancer mortality (% per year)***

|  | Age and stage-specific cervical cancer mortality (% per year) | | |
| --- | --- | --- | --- |
| Age | for localised cervical cancer (LCC) | for regional cervical cancer (RCC) | for distant cervical cancer (DCC) |
| 12–29 years | 0.7 | 13.4 | 42.9 |
| 30–39 years | 0.6 | 8.9 | 41.0 |
| 40–49 years | 0.8 | 11.0 | 46.7 |
| 50–59 years | 1.9 | 10.1 | 52.7 |
| 60–69 years | 4.2 | 17.6 | 54.6 |
| ≥70 years | 11.6 | 28.6 | 70.3 |

* Source: Surveillance, Epidemiology, and End Results (SEER) program. Public-use data (1973–2002), National Cancer Institute, DCCPS, Surveillance Research Program, Cancer Statistics Branch. Released 2005 Apr, based on the November 2004 submission [cited 2006 Mar 13]. (<http://www.seer.cancer.gov>)

**Table A3. Mean number of sexual partners per year by age groups, Estonia ***

|  | Mean number of sexual partners per year | |
| --- | --- | --- |
| Age | Male | Female |
| 13-14 years | 0.00001 | 0.00001 |
| 15-17 years | 0.47 | 0.29 |
| 18 years | 1.87 | 1.14 |
| 19 years | 1.87 | 1.14 |
| 20-24 years | 1.56 | 1.10 |
| 25-26 years | 1.45 | 1.09 |
| 27-29 years | 1.45 | 1.09 |
| 30-34 years | 1.22 | 1.07 |
| 35-39 years | 1.25 | 1.07 |
| 40-44 years | 1.21 | 1.02 |
| 45-49 years | 0.40 | 0.34 |
| 50-54 years | 0.40 | 0.34 |
| 55-59 years | 0.00074 | 0.00063 |
| 60-64 years | 0.00061 | 0.00061 |
| 65-69 years | 0.00061 | 0.00061 |
| 70-74 years | 0.00044 | 0.00044 |
| 75-79 years | 0.00044 | 0.00044 |

* Source: Estonian Health Interview Survey 2006 (<http://www2.tai.ee/ETeU/met_51.pdf>); data modified for model calibration.

**Table A4. Distribution of sexual activity risk groups and corresponding mean numbers of partners per year, Estonia**

|  | Male | | Female | |
| --- | --- | --- | --- | --- |
|  | Percent of the population in the sexual activity risk group | Mean number of sexual partners per year | Percent of the population in the sexual activity risk group | Mean number of sexual partners per year |
| Low (0-1 mean sexual partners per year) | 88% | 0.85 | 95% | 0.80 |
| Medium (2-4 mean sexual partners per year) | 10% | 2.7 | 4.6% | 2.4 |
| High (5 or more mean sexual partners per year) | 2% | 6.0 | 0.4% | 6.0 |

* Source: Estonian Health Interview Survey 2006 (<http://www2.tai.ee/ETeU/met_51.pdf>); data modified for model calibration.

**Table A5. Screening and treatment parameters*, Estonia**

| Parameter | Estimate | Source |
| --- | --- | --- |
| Routine cervical cytology screening, (% per year) | | Estonian Health Insurance Fund data, 2008, Number of women in 2008 who had either PAP smear either in opportunistic or organized screening programme / Number of women in age group |
| 9-14 years | 1% |  |
| 15-19 years | 20% |  |
| 20-24 years | 36% |  |
| 25-29 years | 40% |  |
| 30–34 years | 38% |  |
| 35–39 years | 35% |  |
| 40–44 years | 33% |  |
| 45–49 years | 32% |  |
| 50–54 years | 28% |  |
| 55–59 years | 20% |  |
| 60–64 years | 17% |  |
| 65–69 years | 14% |  |
| 70–74 years | 11% |  |
| 75–79 years | 7% |  |
| 80-84 years | 4% |  |
| 85+ years |  |  |
| What percent of females receive a follow-up screening test after an abnormal PAP smear diagnosis? | 58% | Expert Panel (based on Estonian Health Insurance Fund inquiry, 2008 data) |
| What percent of females receive cervical cancer screening tests at least once every three years? | 72% | Estonian Health Insurance Fund, 2010 |
| Recognize symptoms and seek treatment, % per year | | U.S. data (expert opinion) used in Elbasha et al 2010 *** |
| Localized cervical cancer | 3.8% |  |
| Regional cervical cancer | 18% |  |
| Distant cervical cancer | 90% |  |
| Percent of CIN 1 treated/followed-up | 50% | Expert Panel |
| Percent of CIN 2, CIN 3, CIS treated | 100% | Expert Panel |
| Percent of genital warts treated - males | 31% | Estonian Health Insurance Fund database ** |
| Percent of genital warts treated - females | 75% |  |
| What percent of the female population receives hysterectomy over the course of 1 year by age group? | | Estonian Health Insurance Fund database inquiry (2008 data) |
| 15–24 years | 0.012% |  |
| 25-29 years | 0.078% |  |
| 30–34 years | 0.188% |  |
| 35–39 years | 0.37% |  |
| 40–44 years | 0.815% |  |
| 45–54 years | 0.894% |  |
| ≥55 years | 0.322% |  |

* CIN, cervical intraepithelial neoplasia; CIS, carcinoma in situ

** True prevalence of GW in Estonia is not known. Based on international data, we are assuming approximately 160 per 100,000 persons and treatment rates of 31% and 75% for males/females, respectively, to arrive at actual treatment prevalence of 50 cases per 100,000 males and 123 cases per 100,000 females as recorded in HIF database over 7 year period of 2004-2010.

*** Elbasha EH, Dasbach EJ: Impact of vaccinating boys and men against HPV in the United States. Vaccine 2010; 28, 6858–6867.

**Table A6. Costs of diagnosing and treating HPV* in Estonia (in euros)**

| Parameter | Estimate | Source |
| --- | --- | --- |
| Conventional cytology screening exam including visit cost | 27.36 | Expert panel and Estonian Health Insurance Fund official tariffs ** |
| Colposcopy | 26.14 | Estonian Health Insurance Fund database inquiry, 2007-2009 |
| Biopsy | 12.31 |  |
| CIN 1 treatment | 169.24 |  |
| CIN 2 treatment | 290.76 |  |
| CIN 3 treatment | 508.63 |  |
| Localized cervical cancer treatment | 3,054.86 | Estonian Health Insurance Fund database inquiry, 2007-2009 and expert opinion based allocation by stage |
| Regional cervical cancer treatment | 7,282.34 |  |
| Distant cervical cancer treatment | 2,131.97 |  |
| Genital wart treatment - male | 26.78 | Expert opinion based on Estonian Health Insurance Fund database inquiry in 2008 as to GW treatment specific procedures and 5% imiquimod cream use |
| Genital wart treatment - female | 26.78 |  |
| Vaccine price per dose | 59.0 | Assumption made by Liiv et al [15] in analysis for Estonian Ministry of Social Affairs |

* CIN = cervical intraepithelial neoplasia; DCC = distant cervical cancer; HPV = human papillomavirus; LCC = localized cervical cancer; RCC = regional cervical cancer.

** Estonian Health Insurance Fund tariffs, 2011

**Table A7. Quality of life parameters*, Estonia**

| Parameter | Female | Male | Source |
| --- | --- | --- | --- |
| No condition |  |  |  |
| 12–17 y | 0.93 | 0.93 | Gold, 1998 |
| 18–34 y | 0.91 | 0.92 |  |
| 35–44 y | 0.89 | 0.90 |  |
| 45–54 y | 0.86 | 0.87 |  |
| 55–64 y | 0.80 | 0.81 |  |
| 65–74 y | 0.78 | 0.76 |  |
| >75 y | 0.70 | 0.69 |  |
| CIN 1 | 0.91 | - | Myers, 2004 |
| CIN 2/3, CIS | 0.87 | - | Myers, 2004 |
| Localized cervical cancer | 0.76 | - | Myers, 2004 |
| Regional cervical cancer | 0.67 | - | Myers, 2004 |
| Distant cervical cancer | 0.48 | - | Gold, 1998 |
| Cervical cancer survivor | 0.76 | - | Gold, 1998; Andersen, 1996; Wenzel, 2005 |
| Genital warts | 0.91 | - | Myers, 2004 |

* CIN, cervical intraepithelial neoplasia; CIS, carcinoma in situ; F, females; M, males.

Myers E, Green S, Lipkus I: Patient preferences for health states related to HPV infection: visual analogue scales vs. time trade-off elicitation. Proceedings of the 21st International Papillomavirus Conference. Abstract no. 390.2. Mexico City, Mexico. 2004.

Gold M, Franks P, McCoy K, Fryback D: Toward consistency in cost-utilities analysis: using national measures to create condition-specific values. Med Care 1998, 36, 778–92.

Andersen B: Stress and quality of life following cervical cancer. J Natl Cancer Inst 1996, 21, 65–70.

Wenzel L, DeAlba I, Habbal R, Kluhsman BC, Fairclough D, Krebs L, et al.: Quality of life in long-term cervical cancer survivors. Gynecol Oncol 2005, 97, 310–7. **Table A8. Diagnosis and treatment costs of genital warts – male and female *^,^**, Estonia**

| As per 2009 Estonian Health Insurance Fund data | No | Estonian Health Insurance Fund cost per item, € | Total Cost, € |
| --- | --- | --- | --- |
| Cryotherapies (insurance claim with ICD-10 diagnose code A63) | 131 | € 11.25^a^ | € 1,474 |
| 672 persons treated with Sol Podophyllotoxini 5mg / ml | 672 | € 11.43^b^ | € 7,678 |
| 729 persons treated with 5% imiquimodum cream | 729 | € 15.72^b^ | € 11,463 |
| Subtotal | 1,532 | € 13.46 | € 20,615 |
| Specialist visit | 1 | € 11.02^c^ |  |
| Return specialist visit | Estimated 24% of cases | € 2.28^e^ |  |
| Total estimate per average patient |  | € 26.77*** |  |

* Codes for all medical services and procedures used based on either Estonian Health Insurance Fund-specific procedure (i.e. specialist visit) or Nordic Medico-Statistical Committee (NOMESCO) classification of Surgical Procedures codes: a - Estonian Health Insurance Fund code 7025; b - average cost to Estonian Health Insurance Fund; c - Estonian Health Insurance Fund code 3002; d - Estonian Health Insurance Fund code 3004: € 9.25 × 24% = € 2.28;

** Cost estimate is based on expert opinion as to resource utilization and Estonian Health Insurance Fund unit costs 2011 as shown in the table

*** This cost estimate of € 26.77 is less than average actual Estonian Health Insurance Fund cost for genital warts (ICD-10 diagnose code A63.0) care (€61). Our cost estimate does not include routine sexually transmitted infections screening costs what also often take place during the visit for genital warts treatment, as the benefits of this cost are not included in the model as well.

**Table A9. Costs related to the conventional cytology screening exam, Estonia ***

| code | Name | Mean no. of episodes or % of service utilization | Reimbursement price | Total, € |
| --- | --- | --- | --- | --- |
| 3002 | First specialist physician visit | 1 | € 12.02 | € 12.02 |
| 3004 | Repeat specialist physician visit | 75% | € 9.52 | € 7.14 |
| 66807 | PAP test, normal | 1 | € 7.12 | € 7.12 |
| 66809 | PAP test, pathology | 6% | € 7.12 | € 0.43 |
| 66608 | HPV test with negative result | 2.27%^a^ | € 12.93 | € 0.29 |
| 66612 | HPV test with positive result | 1.02%^b^ | € 25.50 | € 0.26 |
| Total |  |  |  | € 27.36 |

a HPV test in estimated 55% of cases of pathological PAP, 69% of HPV tests are negative: 6% ×55% ×69% = 2.27%

b HPV test in estimated 55% of cases of pathological PAP, 31% of HPV tests are positive; 6% ×55% ×31% = 1.02%

* Cost estimate is based on expert opinion as to resource utilization and Estonian Health Insurance Fund unit costs 2011 as shown in the table

**Table A10. Costs related to colposcopy, Estonia ***

| code | Name | Mean no. of episodes of service utilization | Reimbursement price | Total, € |
| --- | --- | --- | --- | --- |
| 3002 | First specialist physician visit | 1 | 12.02 | 12.02 |
| 7563 | Colposcopy | 1 | 14.12 | 14.12 |
| Total |  |  |  | 26.14 |

* Cost estimate is based on expert opinion as to resource utilization and Estonian Health Insurance Fund unit costs 2011 as shown in the table

**Table A11. Costs related to biopsy, Estonia***

| code | Name | Mean no. of episodes or % of service utilization | Reimbursement price | Total, € |
| --- | --- | --- | --- | --- |
| 7004 | Biopsy | 1 | 2.30 | 2.30 |
| 66800 | Histology | 99% | 8.80 | 8.71 |
| 66608 | HPV test (PCR) | 10% | 12.93 | 1.29 |
| Total |  |  |  | 12.31 |

* Cost estimate is based on expert opinion as to resource utilization and Estonian Health Insurance Fund unit costs 2011 as shown in the table

**Table A12. Diagnosis and treatment costs assumed for CIN1, CIN 2/3, Estonia***

| Disease state cost per episode of care | € per incident case |
| --- | --- |
| CIN 1 | 169.2 |
| CIN2 | 290.8 |
| CIN3 | 508.6 |

* Costs shown are based on inquiry in Estonian Health Insurance Fund database for the period of 2007-2009. Cost per episode of care is calculated as mean value of all Estonian Health Insurance Fund procedures with main diagnose of N87.0, N87.1, N87.2 during 12 months after initial diagnose (records restricted to patients where biopsy or CIN specific treatment was used)

**Table A13. Assumed diagnosis and treatment costs of localized cervical cancer (LCC), regional cervical cancer (RCC) and distant cervical cancer (DCC), Estonia***

| Disease state cost per episode of care | € per incident case |
| --- | --- |
| Localized cervical cancer (LCC) | 3,054.9 |
| Regional cervical cancer (RCC) | 7,282.3 |
| Distant cervical cancer (DCC) | 2,132.0 |

* Costs shown are based on inquiry in Estonian Health Insurance Fund database for the period of 2007-2009. Cost per episode of care is calculated including both Estonian Health Insurance Fund services as well as Estonian Health Insurance Fund reimbursed medicines for patients who received first diagnosis of cervical cancer in 2007. Average costs were allocated by stages as per expert opinion. Sick Leave payments also compensated by Estonian Health Insurance Fund are currently not included in cervical cancer diagnosis and treatment cost estimates.
